# Microstructure-Dependent Corrosion Behavior of Ferritic–Martensitic 17Cr Stainless Steel in CO_2_-Saturated Brine at 230 °C Under High Pressure

**DOI:** 10.3390/ma19132899

**Published:** 2026-07-06

**Authors:** Song He, Zhile Yang, Xuesong Xing, Weiru Zheng, Xijin Xing, Xiaoqi Yue

**Affiliations:** 1 Cnooc Research Institute Co., Ltd. in Beijing, Beijing 100028, China; hesong2@cnooc.com.cn (S.H.); xingxs@cnooc.com.cn (X.X.); zhengwr2@cnooc.com.cn (W.Z.); xingxj2@cnooc.com.cn (X.X.); 2 Institute for Advance Materials and Technology, University of Science and Technology Beijing, Beijing 100083, China; yangzhile2021@outlook.com

**Keywords:** 17Cr stainless steel, CO_2_ corrosion, ferritic–martensitic microstructure, localized corrosion, Raman spectroscopy

## Abstract

**Highlights:**

- Corrosion rate increased markedly with increasing CO_2_ partial pressure at 230 °C.
- Localized corrosion preferentially occurred in martensitic banded regions.
- High local misorientation was associated with localized surface-film destabilization.
- Ferrite grain boundaries became vulnerable at high CO_2_ pressure.
- Raman spectra identified Cr(OH)_3_ as corrosion product, while EDS revealed pressure-dependent elemental enrichment.

**Abstract:**

The corrosion behavior of ferritic–martensitic 17Cr stainless steel in CO_2_-saturated brine was investigated using static autoclave immersion tests in 4.12 wt% NaCl solution at 230 °C under CO_2_ partial pressures of 6.36, 18.28, and 24.57 MPa. The calculated in situ pH values obtained using the OLI System were 3.79, 3.55, and 3.49, respectively. Corrosion morphology, microstructural evolution, and corrosion products were characterized by SEM, EDS, EBSD, and Raman spectroscopy. The average mass-loss corrosion rate increased from 0.138 ± 0.0221 mm/year at 6.36 MPa pCO_2_ to 0.326 ± 0.0142 mm/year at 24.57 MPa pCO_2_. Although the specimens did not show severe macroscopic pitting, localized attack preferentially occurred in fine-grained martensitic banded regions. EBSD analysis revealed that these regions exhibited higher local misorientation and defect density, which may reduce the stability of Cr-rich surface films. Raman spectra identified Cr(OH)_3_ in the corrosion products, and the Cr(OH)_3_ signal became more evident with increasing CO_2_ partial pressure. The results indicate that, under fixed temperature and salinity, the corrosion behavior of 17Cr stainless steel is governed by CO_2_ partial pressure and microstructural heterogeneity.

## 1. Introduction

With the continuous expansion of energy exploitation toward deep wells, deep-sea reservoirs, and elevated-temperature geothermal systems, structural materials are increasingly exposed to extremely harsh service environments characterized by elevated temperature, high pressure, high salinity, and CO_2_-rich media. Under such conditions, corrosion degradation becomes one of the major factors limiting the reliability and service life of oil and gas production equipment. In particular, the synergistic interaction between temperature, pressure, chloride ions, and dissolved CO_2_ can significantly accelerate the deterioration of metallic materials, leading to severe uniform corrosion, localized corrosion, stress corrosion cracking, and even catastrophic failure of critical components [1,2,3]. Therefore, understanding the corrosion behavior and degradation mechanisms of corrosion-resistant alloys under extreme environments has become an important topic in the field of materials corrosion and protection.

Martensitic stainless steels have been widely employed in tubing, casings, valves, and downhole tools in oil and gas production owing to their favorable combination of high mechanical strength, moderate corrosion resistance, and relatively low cost compared with duplex or nickel-based alloys [4,5]. Among them, Cr-containing martensitic stainless steels, such as 13Cr and 17Cr grades, exhibit enhanced resistance against CO_2_ corrosion because the Cr-enriched passive film can effectively suppress anodic dissolution under mild conditions [6]. However, when the service temperature exceeds approximately 200 °C and the pressure reaches several megapascals, the corrosion resistance of martensitic stainless steels deteriorates markedly. Numerous field failures have demonstrated that elevated-temperature and elevated-pressure environments can destabilize the passive film and promote aggressive localized corrosion, especially in chloride-containing media [7,8]. Consequently, the corrosion behavior and failure mechanisms of martensitic stainless steels under extreme geothermal and oilfield environments remain challenging issues requiring systematic investigation.

The corrosion process in elevated-temperature CO_2_-containing environments is highly complex. Dissolved CO_2_ undergoes hydration and dissociation reactions in aqueous solution, generating H_2_CO_3_, HCO_3^−^_, and CO_3^2−^_ species, which reduce the solution pH and accelerate electrochemical reactions on the metal surface [9]. Simultaneously, high concentrations of NaCl further aggravate corrosion by destabilizing the passive film through chloride adsorption and penetration mechanisms. Chloride ions can induce local passive film breakdown and initiate pitting or crevice corrosion, particularly at microstructural heterogeneities such as grain boundaries, martensite laths, retained austenite regions, and secondary phase interfaces [10,11]. Moreover, elevated temperature and pressure can alter the transport kinetics of ionic species, corrosion product precipitation behavior, and passive film composition, thereby affecting the protectiveness and stability of corrosion scales [12].

Previous studies have investigated the influence of environmental parameters on the corrosion behavior of Cr-containing martensitic stainless steels. Recent work has shown that CO_2_ partial pressure, chloride concentration, flow condition, and autoclave testing parameters can strongly affect corrosion-product formation and localized corrosion in 13Cr, super 13Cr, and 15Cr martensitic stainless steels [13,14,15,16,17,18,19,20,21]. These studies provide important guidance for understanding corrosion scales formed in CO_2_-containing geothermal and oilfield environments. However, the response of ferritic–martensitic 17Cr stainless steel to increasing CO_2_ partial pressure at a fixed elevated temperature and salinity remains insufficiently clarified.

For 17Cr ferritic–martensitic stainless steel, the relationship between ferrite/martensite banding, grain-boundary characteristics, corrosion-product chemistry, and localized corrosion behavior remains insufficiently understood under CO_2_–Cl^−^ environments at 230 °C. In particular, phase-selective attack has rarely been correlated with EBSD-derived local misorientation and corrosion-product identification in the same study [22,23]. Because the present work does not include phase-resolved electrochemical testing, the mechanistic discussion is framed as evidence-supported interpretation rather than direct proof of the electrochemical behavior of each phase.

Therefore, the present work focuses on a single-factor assessment of CO_2_ partial pressure for ferritic–martensitic 17Cr stainless steel in 4.12 wt% NaCl solution at 230 °C. The temperature and chloride concentration were kept constant, while the CO_2_ partial pressure was varied from 6.36 to 24.57 MPa to evaluate its effect on mass-loss corrosion rate, corrosion morphology, and corrosion-product formation. Surface morphology observation, cross-sectional analysis, EBSD, EDS, and Raman spectroscopy were combined to clarify the correlation between ferrite/martensite microstructural heterogeneity and localized corrosion. The results provide guidance for applying 17Cr stainless steel in CO_2_-containing elevated-pressure geothermal and oilfield environments.

## 2. Materials and Methods

### 2.1. Materials

**Figure 1:**
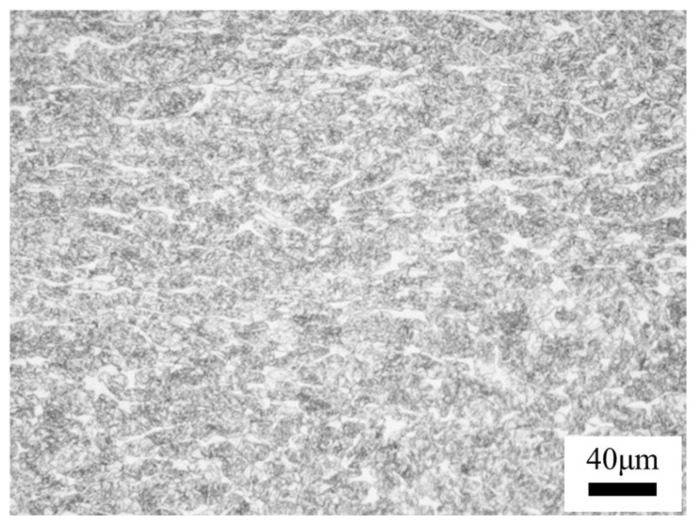
Metallographic structure of ferritic–martensitic 17Cr stainless steel.

The investigated material was a commercial 17Cr stainless steel tubing steel widely used in oilfield applications. Its nominal chemical composition is listed in Table 1. The initial microstructure of the material is shown in Figure 1. The commercial 17Cr stainless steel mainly consisted of ferrite and martensite phases, with a small amount of retained austenite detected by EBSD (Oxford Instruments, Oxford, UK).

Specimens with dimensions of 10 mm × 30 mm × 3 mm were machined for the elevated-temperature and elevated-pressure corrosion tests. Prior to corrosion exposure, the specimen surfaces were sequentially ground using 400# and 800# SiC abrasive papers to remove machining marks and obtain a smooth surface finish. Subsequently, the specimens were ultrasonically cleaned in anhydrous ethanol for 15 min to remove residual contaminants and grease, followed by drying with warm air and storage in a desiccator before testing.

### 2.2. Elevated-Temperature and High-Pressure Corrosion Tests

**Figure 2:**
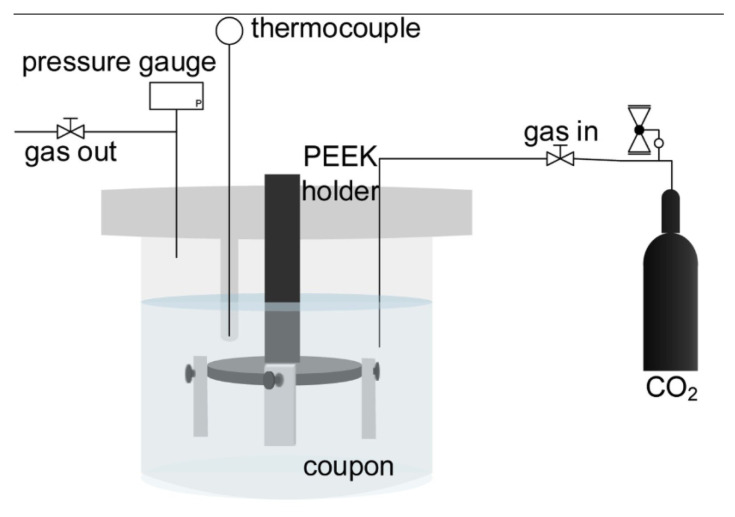
Schematic diagram of the elevated-temperature and high-pressure static corrosion test system.

Static corrosion experiments were carried out using an elevated-temperature and high-pressure autoclave system equipped with a reactor vessel, heating system, pressure control system, temperature control system, and safety protection system, as schematically shown in Figure 2. The prepared specimens were mounted on a specimen holder and immersed in a deoxygenated 4.12 wt% NaCl solution prepared from deionized water and analytical-grade NaCl. The NaCl concentration was selected to simulate a high-salinity CO_2_-containing oilfield/geothermal brine environment.

Before the corrosion tests, the reactor was sealed and purged with CO_2_ gas three times to eliminate residual oxygen inside the autoclave. During each purging cycle, CO_2_ was introduced until the pressure reached 2 MPa and then slowly released to atmospheric pressure. After oxygen removal, CO_2_ was continuously injected until the desired partial pressure at room temperature was achieved. The heating system was then activated to raise the reactor temperature to 230 °C, while the total pressure was adjusted to the required experimental condition. The three CO_2_ partial pressures were selected to represent progressively increasing CO_2_ severity while keeping temperature, NaCl concentration, and exposure time constant. Because the room-temperature solution was only CO_2_-saturated before heating and pressurization, room-temperature pH was not used to represent the actual corrosion environment. Instead, the in situ pH values at 230 °C were calculated using the OLI System 12.5 (OLI Systems, Inc., Parsippany, NJ, USA) as 3.79, 3.55, and 3.49 for the 9.71, 24.08, and 29.70 MPa total-pressure conditions, respectively. The corrosion test duration was 120 h. During the entire exposure process, temperature and pressure were continuously monitored to ensure stable testing conditions. The detailed experimental conditions are summarized in Table 2.

### 2.3. Corrosion Rate Measurement

**Figure 3:**
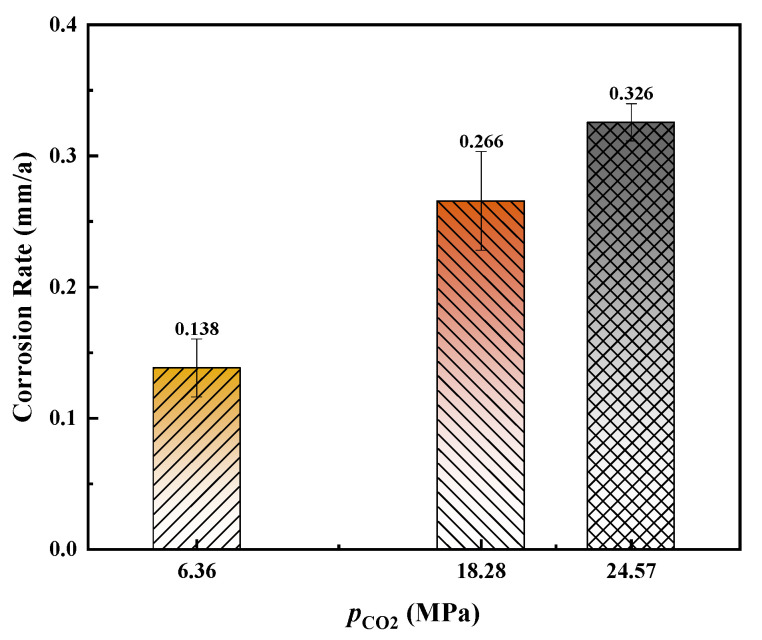
Average mass-loss corrosion rate of ferritic–martensitic 17Cr stainless steel at 230 °C under different CO_2_ partial pressures. Error bars represent standard deviation from three replicate specimens.

After corrosion testing, the autoclave was naturally cooled to room temperature, and the internal pressure was gradually released to atmospheric pressure. The specimens were removed, gently rinsed, dried, and weighed after exposure. Corrosion products were then removed by immersion in 20% nitric acid solution, followed by rinsing, drying, and final weighing. The mass loss was determined from the difference between the initial mass and the final mass after corrosion-product removal. Three replicate specimens were tested for each condition, and the error bars in Figure 3 represent the standard deviation. The average mass-loss corrosion rate was calculated according to the following equation:

V = 87,600 × Δm/(ρ × S × t)(1)

where V is the average mass-loss corrosion rate (mm/year, millimetres per year), Δm is the mass loss before exposure and after corrosion-product removal (g), ρ is the density of the tested material (7.75 g/cm^3^), S is the exposed surface area of the specimen (cm^2^), and t is the exposure time (h). Because localized and phase-selective dissolution contribute to the measured mass loss, the calculated value represents an average corrosion rate rather than a true uniform-thinning rate.

### 2.4. Microstructural and Corrosion Characterization

The surface and cross-sectional morphologies of the corroded specimens were characterized using scanning electron microscopy (SEM, Regulus 8100 from Hitachi High-Tech Corporation, Tokyo, Japan and Zeiss Merlin Compact from Oberkochen, Germany), to evaluate corrosion damage features and corrosion morphology evolution. SEM observations were performed at accelerating voltages of 15–20 kV. Energy-dispersive spectroscopy (EDS) was employed for qualitative and quantitative analysis of corrosion products and corrosion interfaces to determine elemental distribution characteristics and provide evidence for corrosion mechanism analysis. Raman spectroscopy was further used to identify corrosion-product phases.

Electron backscatter diffraction (EBSD) characterization was further performed to investigate the microstructural features of the matrix, including grain size distribution, phase constitution (ferrite bcc and austenite fcc), kernel average misorientation (KAM), and grain boundary characteristics. The EBSD step size was 0.2 μm. These analyses were used to evaluate the relationship between microstructural heterogeneity and corrosion behavior under elevated-temperature and high-pressure environments.

## 3. Results

### 3.1. Average Mass-Loss Corrosion Rate

Figure 3 presents the variation in the average mass-loss corrosion rate of the ferritic–martensitic 17Cr stainless steel as a function of CO_2_ partial pressure at 230 °C. The average corrosion rate was 0.138 ± 0.0221 mm/year under a CO_2_ partial pressure of 6.36 MPa. When the CO_2_ partial pressure increased to 18.28 MPa, the corrosion rate nearly doubled, reaching 0.266 ± 0.0376 mm/year. With a further increase in CO_2_ partial pressure to 24.57 MPa, the corrosion rate continued to rise to 0.326 ± 0.0142 mm/year. The results indicate that increasing CO_2_ partial pressure significantly accelerates the mass loss of the ferritic–martensitic 17Cr stainless steel under the present elevated-temperature conditions. This behavior is mainly associated with the enhanced dissolution of CO_2_ and the increased formation of carbonic acid species in the solution, which promote electrochemical reactions and weaken the stability of the Cr-rich surface film.

**Figure 4:**
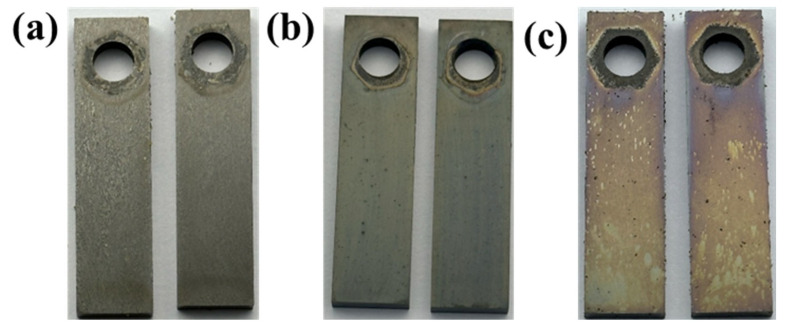
Macroscopic photographs of the specimens after corrosion experiments: (**a**) 230–6.36 MPa pCO_2_; (**b**) 230–18.28 MPa pCO_2_; (**c**) 230–24.57 MPa pCO_2_.

The macroscopic morphologies of the specimens after corrosion immersion tests are shown in Figure 4. After exposure, the metallic luster of the specimens disappeared completely, and the surfaces were covered by dark corrosion products. The corrosion product layer was relatively uniformly distributed over the entire surface, without obvious signs of severe macroscopic pitting or crevice corrosion. These observations indicate that macroscopic surface coverage was relatively uniform, although subsequent microscopic characterization shows that microstructural heterogeneity strongly influenced local corrosion propagation.

**Figure 5:**
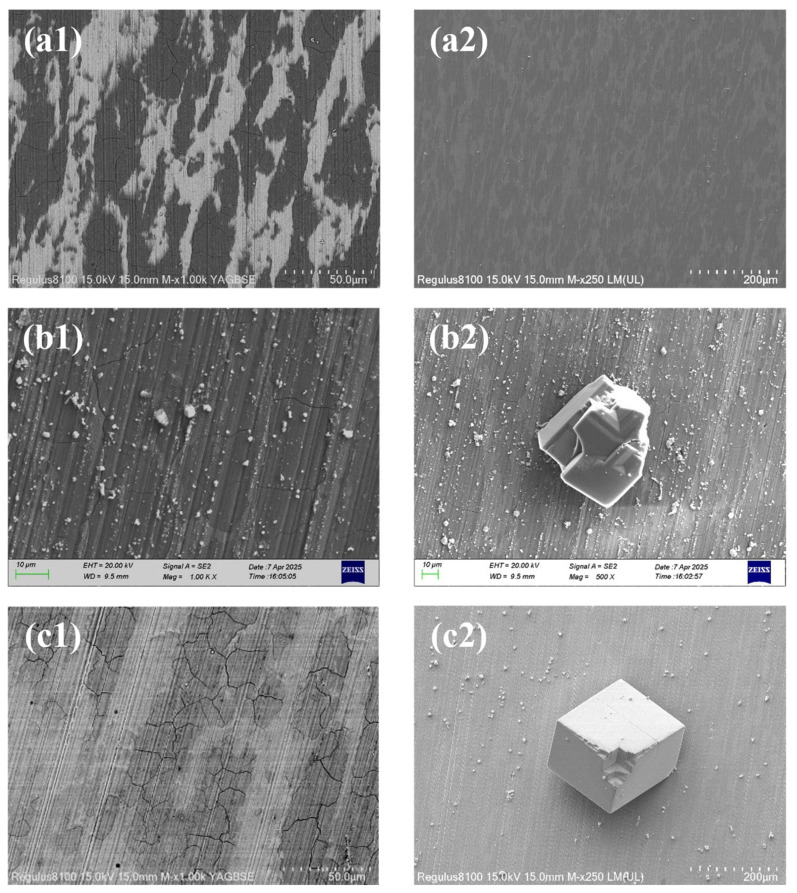
Morphology of corrosion products on the surface of ferritic–martensitic 17Cr stainless steel: (**a1**–**c1**) are BES images in different pCO_2_ under 230 °C (**a1**) 230–6.36 MPa pCO_2_; (**b1**) 230–18.28 MPa pCO_2_; (**c1**) 230–24.57 MPa pCO_2_. (**a2**–**c2**) are the distribution characteristics of the surface corrosion products. (**a2**) 230–6.36 MPa pCO_2_; (**b2**) 230–18.28 MPa pCO_2_; (**c2**) 230–24.57 MPa pCO_2_.

Figure 5 shows the surface micro-morphologies of the ferritic–martensitic 17Cr stainless steel after immersion at 230 °C under different CO_2_ partial pressures. Higher-magnification images in Figure 5(a1–c1) reveal distinct corrosion tendencies associated with different CO_2_ partial pressures. In particular, the backscattered electron (BSE) images in Figure 5(a1,c1) exhibit corrosion product distributions closely correlated with the banded microstructure shown in Figure 1. Regions enriched with oxygen appeared darker in the BSE images, indicating preferential formation or accumulation of oxide/hydroxide-rich corrosion products along specific microstructural regions.

The low-magnification morphologies shown in Figure 5(a2–c2) further illustrate the distribution characteristics of the surface corrosion products. Under CO_2_ partial pressures of 18.28 MPa and 24.57 MPa at 230 °C, distinct cubic precipitated products were observed on the specimen surfaces. Similar cubic corrosion products have been reported in CO_2_-containing brines and are commonly associated with Fe-containing carbonate products such as FeCO_3_ or mixed corrosion scales, although EDS alone cannot unambiguously identify the phase. The appearance of these cubic products suggests that increasing CO_2_ partial pressure affects the nucleation and growth behavior of corrosion products.

### 3.2. Microstructure-Related Localized Corrosion Behavior

**Figure 6:**
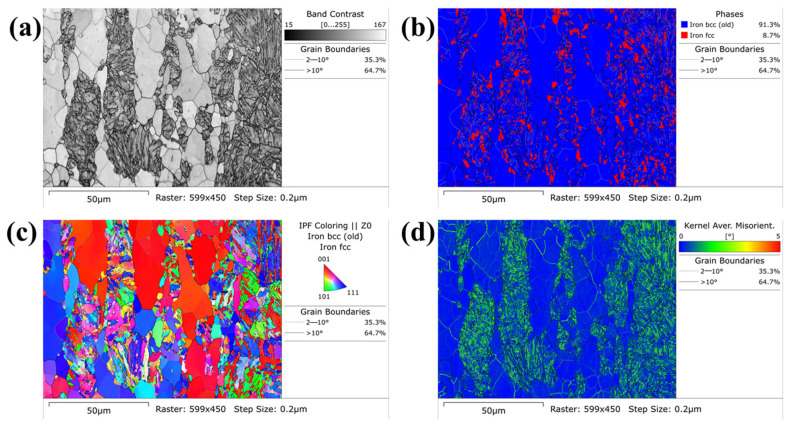
EBSD map of ferritic–martensitic 17Cr stainless steel: (**a**) band contrast and grain-boundary map; (**b**) phase map; (**c**) IPF map; (**d**) KAM map.

Figure 6 presents the EBSD characterization results of the ferritic–martensitic 17Cr stainless steel. The banded microstructure can be divided into ferrite bands with relatively coarse grains and prior austenite bands mainly composed of martensite and retained austenite, as shown in Figure 6a. The EBSD phase map indicates that the steel contains ferrite/martensite-dominated bcc regions and approximately 8.7% retained austenite, as shown in Figure 6b. Therefore, the term ferritic–martensitic 17Cr stainless steel is used in the revised manuscript to avoid implying several primary phases. In addition, the grain size within the prior austenite bands was significantly refined compared with that of the ferrite bands, and no obvious preferred crystallographic orientation was observed, as shown in Figure 6c.

Figure 6d further reveals the relationship between local misorientation and microstructural distribution. Regions with high misorientation values were mainly concentrated within the prior austenite lath structures, whereas in the ferrite bands, high misorientation was primarily localized near grain boundaries. The higher local misorientation in martensitic regions indicates a greater density of lattice defects, residual strain, and crystallographic distortion. These features may increase local electrochemical activity and reduce the stability of Cr-rich surface films during corrosion exposure; however, this interpretation is based on microstructural correlation and should not be regarded as direct phase-resolved electrochemical proof.

**Figure 7:**
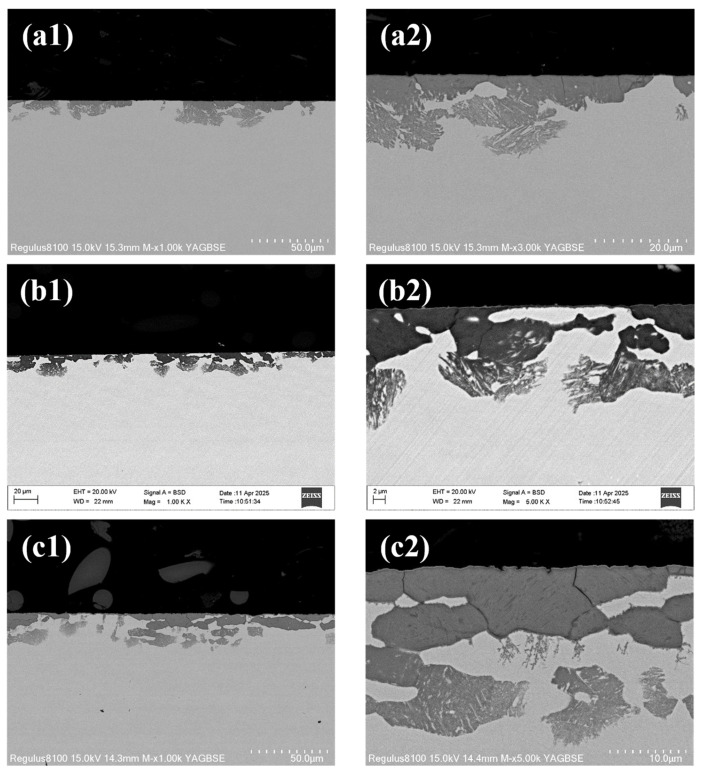
Cross-sectional morphology of ferritic–martensitic 17Cr stainless steel: (**a1**–**c1**) are BES images in different pCO_2_ under 230 °C (**a1**) 230–6.36 MPa pCO_2_; (**b1**) 230–18.28 MPa pCO_2_; (**c1**) 230–24.57 MPa pCO_2_. (**a2**–**c2**) are BSE images at higher magnification. (**a2**) 230–6.36 MPa pCO_2_; (**b2**) 230–18.28 MPa pCO_2_; (**c2**) 230–24.57 MPa pCO_2_.

The cross-sectional morphologies of the corroded specimens after exposure at 230 °C under different CO_2_ partial pressures are shown in Figure 7. The low-magnification images in Figure 7(a1–c1) illustrate the inward propagation of corrosion from the surface into the substrate, indicating a localized corrosion tendency closely associated with the heterogeneous microstructure. Higher-magnification observations in Figure 7(a2–c2) demonstrate that localized corrosion preferentially occurred within the fine-grained banded regions, which correspond well to the prior austenite bands identified in the EBSD analysis shown in Figure 6. Figure 7 is used to show the morphology and location of localized attack; statistically reliable corrosion-product thickness measurements were performed and summarised in Table 3.

At 230 °C, the outermost prior austenite grains were strongly attacked by corrosion, while in the subsurface regions, preferential attack mainly occurred along martensitic laths. This phenomenon suggests that the martensitic regions were more susceptible to localized corrosion than ferrite under the present CO_2_-containing environments. The preferential dissolution of martensitic laths may be related to their high defect density, residual stress, and local compositional heterogeneity introduced during phase transformation. When the CO_2_ partial pressure increased to 24.57 MPa, as shown in Figure 7(c2), corrosion attack also propagated along ferrite grain boundaries, indicating that the aggressive environment further promoted grain-boundary corrosion in the ferrite region.

**Figure 8:**
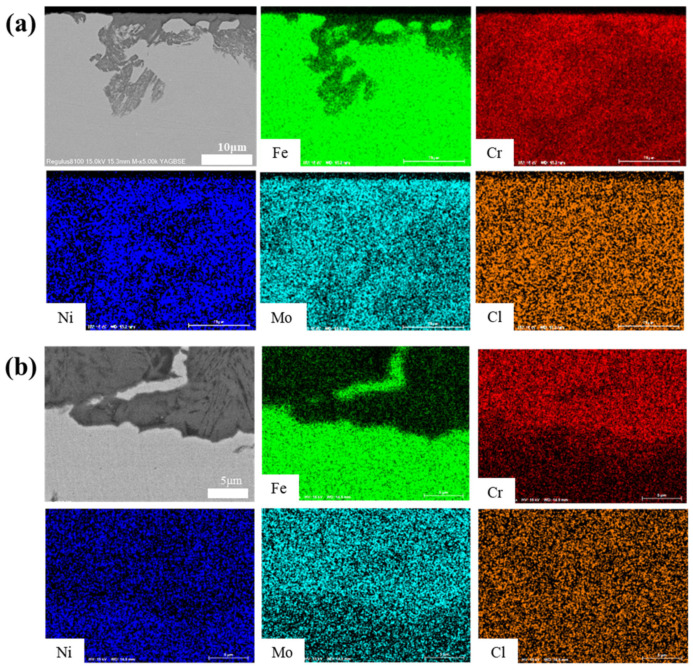
EDS elemental distribution of corrosion products on ferritic–martensitic 17Cr stainless steel: (**a**) 230–6.36 MPa pCO_2_; (**b**) 230–24.57 MPa pCO_2_.

Figure 8 shows the EDS elemental distribution maps of corrosion products formed under 230–6.36 MPa pCO_2_ and 230–24.57 MPa pCO_2_ conditions. Under the lower CO_2_ partial pressure of 6.36 MPa, the corrosion products exhibited Fe depletion and Cr enrichment, indicating that the corrosion layer contained Cr-rich corrosion products. The enrichment of Cr suggests selective dissolution of Fe during corrosion and subsequent enrichment of Cr-containing oxide/hydroxide products. However, EDS provides elemental information only and cannot independently determine the crystalline or molecular phase.

In contrast, under the higher CO_2_ partial pressure of 24.57 MPa, enrichment of Mo and depletion of Ni were additionally observed within the corrosion products. The Mo enrichment may be associated with the preferential retention of Mo-containing oxide/hydroxide products due to their relatively high thermodynamic stability under elevated-temperature corrosion conditions. Meanwhile, the depletion of Ni indicates that Ni-containing regions were less stable in the aggressive high-CO_2_ environment, possibly due to selective dissolution or inhibited incorporation into the corrosion product layer. These compositional variations indicate that increasing CO_2_ partial pressure alters the chemical distribution within the corrosion products, thereby influencing the localized corrosion behavior of the ferritic–martensitic stainless steel.

**Figure 9:**
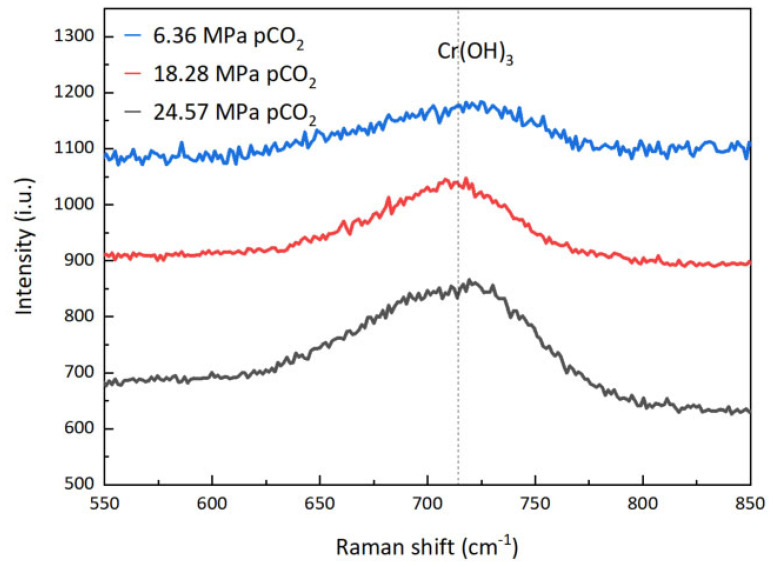
Raman spectra of corrosion products formed on ferritic–martensitic 17Cr stainless steel at 230 °C under different CO_2_ partial pressures.

Raman spectroscopy was used to further identify the corrosion-product phase, as shown in Figure 9. A characteristic Cr(OH)_3_ signal was detected in the corrosion products. The signal became more evident with increasing CO_2_ partial pressure, which is consistent with the thickening/enrichment of Cr-containing corrosion products under more aggressive CO_2_ conditions.

## 4. Discussion

The corrosion behavior of the ferritic–martensitic 17Cr stainless steel under CO_2_-saturated brine at 230 °C was strongly influenced by CO_2_ partial pressure and microstructural heterogeneity. The experimental results demonstrated that increasing CO_2_ partial pressure significantly accelerated the mass-loss corrosion process. At 230 °C, the average corrosion rate increased from 0.138 ± 0.0221 mm/year at 6.36 MPa pCO_2_ to 0.326 ± 0.0142 mm/year at 24.57 MPa pCO_2_, indicating that the aggressive effect of dissolved CO_2_ became increasingly dominant under elevated pressure conditions.

The increase in corrosion rate with increasing CO_2_ partial pressure can primarily be attributed to the enhanced dissolution and ionization of CO_2_ in the aqueous environment. Under elevated-temperature and high-pressure conditions, CO_2_ dissolves into the electrolyte and forms carbonic acid species through hydration and dissociation reactions, thereby decreasing the calculated in situ pH from 3.79 to 3.49 and accelerating anodic dissolution reactions on the steel surface [24,25]. The main reactions can be expressed as CO_2_ + H_2_O ⇌ H_2_CO_3_, H_2_CO_3_ ⇌ H^+^ + HCO_3^−^_, Fe → Fe^2+^ + 2e^−^, and 2H^+^ + 2e^−^ → H_2_. Chromium can also dissolve locally as Cr → Cr^3+^ + 3e^−^, followed by hydrolysis/precipitation reactions such as Cr^3+^ + 3H_2_O → Cr(OH)_3_ + 3H^+^ [18,19,21]. Therefore, Cr(OH)_3_ formation does not require dissolved oxygen; oxygen in the product originates from water and carbonate species. The Raman spectra in Figure 9 confirm the presence of Cr(OH)_3_ in the corrosion products.

The macroscopic observations indicated that corrosion products formed relatively uniformly on the specimen surface without severe macroscopic pitting. However, microscopic characterization revealed pronounced microstructure-dependent localized corrosion behavior. The BSE observations demonstrated that the corrosion products were distributed preferentially along the banded microstructure, indicating that local corrosion susceptibility varied between different microstructural regions. EBSD analysis further confirmed that the prior austenite bands mainly consisted of fine martensitic laths with relatively high local misorientation, while ferrite regions exhibited coarser grains and lower internal distortion.

The preferential corrosion of martensitic regions is interpreted to be associated with their higher defect density, residual stress, and crystallographic distortion introduced during martensitic transformation. High local misorientation generally corresponds to increased dislocation density and lattice strain, which may destabilize Cr-rich surface films and enhance local electrochemical reactivity [26,27]. Furthermore, the refined lath structure of martensite increases the density of phase interfaces and high-energy boundaries, providing preferential pathways for corrosive species penetration. Because phase-resolved electrochemical data were not obtained, this explanation should be regarded as a plausible mechanism supported by EBSD-corrosion morphology correlation rather than definitive proof.

As the CO_2_ partial pressure increased to 24.57 MPa, corrosion propagation extended to ferrite grain boundaries. Grain boundaries generally possess higher energy and enhanced diffusion kinetics compared with grain interiors, making them more susceptible to corrosion attack under severe environments [28]. The transition from preferential martensitic attack to additional ferrite grain-boundary corrosion suggests that increasing CO_2_ partial pressure gradually reduced the corrosion-resistance difference between ferrite and martensitic regions.

The EDS and Raman results further revealed changes in corrosion-product chemistry with increasing CO_2_ partial pressure. Under relatively low pCO_2_ conditions, the corrosion products were characterized by Fe depletion and Cr enrichment, indicating the formation of Cr-rich oxide/hydroxide products. Raman spectroscopy identified Cr(OH)_3_ as a corrosion product, and the Cr(OH)_3_ signal became more evident with increasing CO_2_ partial pressure [29]. At higher pCO_2_ conditions, Mo enrichment and Ni depletion became evident within the corrosion products. The enrichment of Mo may indicate the preferential retention of Mo-containing oxide/hydroxide products due to their superior thermodynamic stability under elevated-temperature corrosion conditions [30]. In contrast, the depletion of Ni suggests that Ni-containing regions were less stable and more susceptible to selective dissolution in the aggressive CO_2_-rich environment.

In addition, cubic precipitated corrosion products observed under high pCO_2_ conditions may be associated with the crystallization of Fe-containing carbonate products, such as FeCO_3_, or mixed oxide/hydroxide phases formed during elevated-temperature CO_2_ corrosion reactions. Similar cubic morphologies and multilayer corrosion products containing FeCO_3_, FeCr_2_O_4_, and Cr(OH)_3_ have been reported for Cr-containing martensitic stainless steels exposed to CO_2_-containing brines [18,19,20,21,31]. Nevertheless, because the present Raman result mainly confirms Cr(OH)_3_ and no XRD/XPS data were obtained, the identification of cubic products as FeCO_3_ remains a literature-supported inference rather than direct phase identification.

Overall, under fixed temperature and salinity, the corrosion degradation of ferritic–martensitic 17Cr stainless steel was controlled by increasing CO_2_ partial pressure and microstructural heterogeneity. The localized preferential dissolution of martensitic regions, combined with reduced stability of Cr-rich surface films under elevated pCO_2_ conditions, governed the corrosion evolution behavior of the material.

## 5. Conclusions

The average mass-loss corrosion rate of ferritic–martensitic 17Cr stainless steel increased significantly with increasing CO_2_ partial pressure at 230 °C. The corrosion rate increased from 0.138 ± 0.0221 mm/year at 6.36 MPa pCO_2_ to 0.326 ± 0.0142 mm/year at 24.57 MPa pCO_2_, indicating that elevated CO_2_ pressure strongly accelerated the corrosion process under the fixed salinity and temperature used in this study. The corrosion behavior was governed by the combined effects of CO_2_-induced reduction in surface-film stability and microstructural heterogeneity, particularly the preferential attack of martensitic lath regions.

After corrosion exposure, the specimen surfaces were covered by dark corrosion products without obvious macroscopic localized corrosion. However, microscopic observations revealed distinct microstructure-dependent localized corrosion behavior associated with the banded microstructure. Localized corrosion preferentially initiated and propagated within the martensitic regions, which was associated with higher defect density and reduced stability of the Cr-rich surface film. Raman spectroscopy identified Cr(OH)_3_ in the corrosion products, and EDS showed Cr enrichment and Fe depletion under low pCO_2_ conditions. Under high pCO_2_ conditions, additional Mo enrichment and Ni depletion were observed, suggesting that increasing CO_2_ pressure altered the composition and stability of corrosion products.

## Figures and Tables

**Table 1:** Chemical composition of the investigated 17Cr stainless steel (wt%).

C	Cr	Ni	Mo	Si	Mn	P	S	Fe
0.06	17.2	2.1	0.8	0.4	0.5	0.02	0.01	Bal.

**Table 2:** Elevated-temperature and high-pressure corrosion simulation experimental conditions.

Temperature (℃)	Total Pressure (Mpa)	CO_2_ Partial Pressure (MPa)	Solution	Gas	Test Period (h)
230	9.71	6.36	4.12 wt% NaCl	CO_2_	120
230	24.08	18.28	4.12 wt% NaCl	CO_2_	120
230	29.70	24.57	4.12 wt% NaCl	CO_2_	120

**Table 3:** Cross-sectional corrosion-affected thickness and maximum local penetration depth measured from SEM images. Values should be reported as mean ± standard deviation when replicate measurements are available.

pCO_2_ (MPa)	Mean Corrosion-Affected Thickness (μm)	Maximum Local Penetration Depth (μm)	Number of Measured Positions
6.36	17.7	24.5	5
18.28	19.5	27.5	5
24.57	27.2	30.5	5

## Data Availability

The original contributions presented in this study are included in the article. Further inquiries can be directed to the corresponding author.
